# Management of Kounis syndrome: two case reports

**DOI:** 10.1186/s13256-017-1310-7

**Published:** 2017-05-23

**Authors:** Majdi Omri, Hajer Kraiem, Olfa Mejri, Mounir Naija, Naoufel Chebili

**Affiliations:** 1 Faculty of Medicine Ibn El Jazzar of Sousse, Sousse, Tunisia; 2 Emergency Medical Service (SAMU 03), Sahloul University Hospital, Route de la ceinture, 4054 Sousse, Tunisia; 3 Medical Intensive Care Unit, Sahloul University Hospital, Sousse, Tunisia

**Keywords:** Kounis syndrome, Acute coronary syndrome, Anaphylaxis, Management, Case report

## Abstract

**Background:**

Kounis syndrome corresponds to the occurrence of myocardial injury following an allergic insult. This syndrome is infrequent, and is not well known. In consequence, it is usually misdiagnosed leading to inappropriate treatment. The current literature is limited to case studies and there are no international recommendations concerning this topic.

**Case presentation:**

We discussed, through two case reports, the clinical presentation and the management of a 60-year-old North African man and a 45-year-old North African man presenting with chest pain suggesting acute coronary syndrome following anaphylactic reaction. Triggering factors were a drug in the first case and herbal dermal exposure in the second. A clinical examination and electrocardiogram revealed anaphylactic reaction associated with myocardial infarction. Appropriate management of these two life-threatening conditions allowed an improvement in our patients’ condition and their transfer to specialized units.

**Conclusions:**

Although Kounis syndrome is a rare phenomenon, physicians should be aware of its physiopathological mechanisms in order to treat it appropriately. The difficulty lies in the fact that the treatment of either of the two associated entities may worsen the other injury.

**Electronic supplementary material:**

The online version of this article (doi:10.1186/s13256-017-1310-7) contains supplementary material, which is available to authorized users.

## Background

Kounis syndrome (KS) is defined as the occurrence of an acute coronary syndrome (ACS) concomitantly with hypersensitivity reactions triggered by an allergenic event and was first described by Kounis and Zavrasin in 1991 as an allergic angina syndrome [1]. This syndrome is not rare, but is often misdiagnosed leading to inappropriate treatment. Despite its specificities, this topic has rarely been reported in the literature and it has not been discussed in the international recommendations for cardiology.

The purpose of this paper is to explain the special features of management of ACS triggered by drug-related anaphylaxis in the first case and herbal allergy in the second.

## Case presentation

### Case 1

A 60-year-old North African man presented to a primary care hospital with chest pain and an altered mental status. He had a 12-year history of type 2 diabetes and hypertension. His history also included ischemic stroke 4 years ago and he was allergic to penicillin. His medication included captopril, furosemide, and metformin. He was a non-tobacco smoker and had no family history of coronary artery disease.

He took 1000 mg of amoxicillin in automedication for a dry cough. He immediately experienced discomfort and chest pain. He presented to our emergency department 30 minutes later with dizziness, pruritus, warmth, flushing, and dyspnea associated with retrosternal oppression graded 7/10 on a pain scale.

**Fig. 1:**
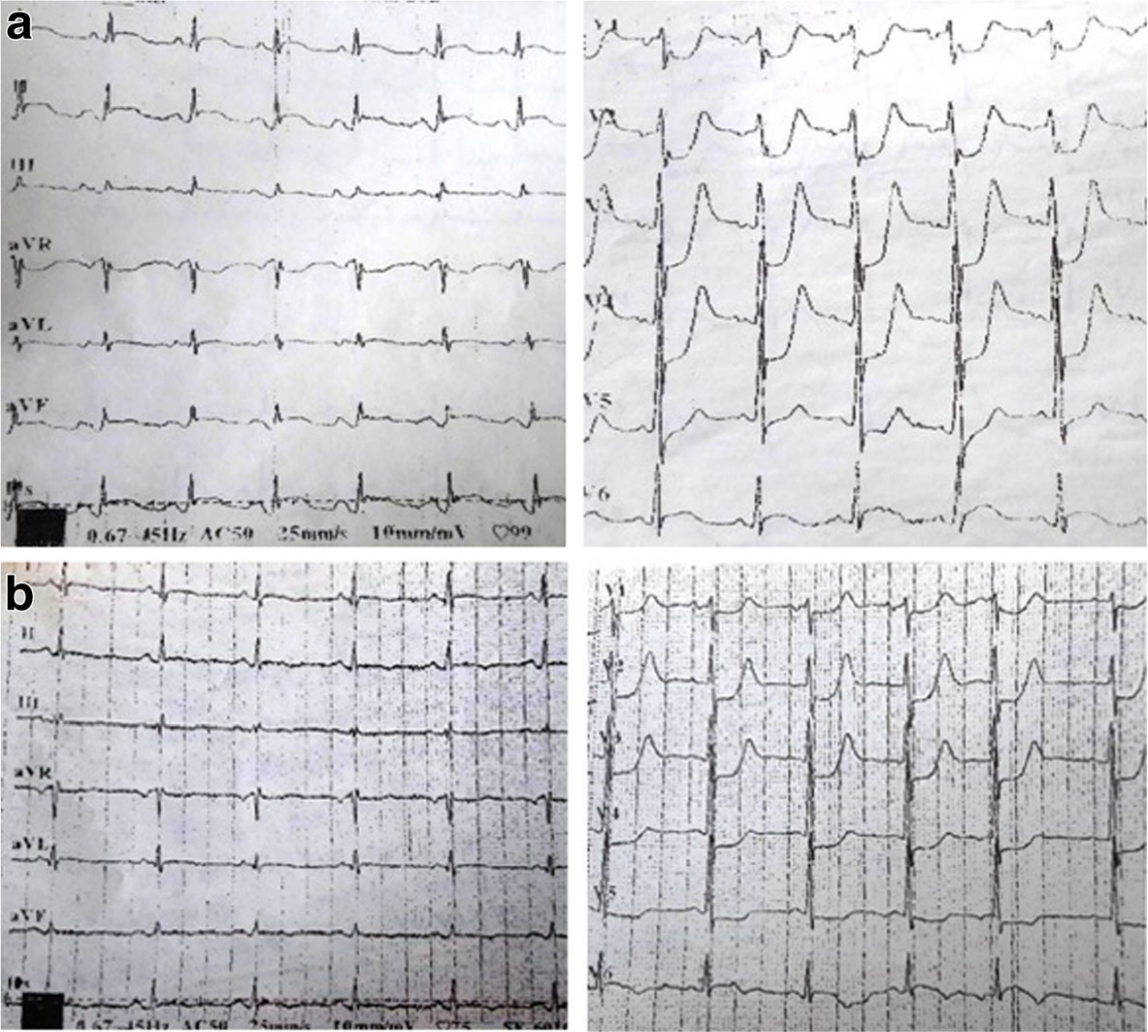
**a** Patient’s initial electrocardiogram showing ST elevation in the inferior wall with reciprocal change in anterolateral leads. **b** Patient’s electrocardiogram after fibrinolysis showing regression of ST elevation in inferior wall

At presentation, he had an altered mental status with a Glasgow Coma Scale (GCS) of 14/15 and had generalized urticaria. His vital signs were stable. An electrocardiogram (ECG) showed sinus rhythm and signs of myocardial injury with significant ST segment elevation of 3 mm in II, III, aVF, V7, and V8 leads and a reciprocal change in the anterolateral wall (Fig. 1a).

In the light of these clinical and electrical findings, the diagnosis of ST segment elevation myocardial infarction concomitant with anaphylactic reaction was strongly suspected.

Dexamethasone and diphenhydramine were intravenously administered in association with anti-ischemic treatment: heparin (50 mg intravenously administered), aspirin (250 mg intravenously administered) and clopidogrel (300 mg administered orally). Fibrinolysis was performed successfully using intravenously administered streptokinase. Our patient’s symptoms gradually improved and his ECG showed that the ST segment elevation returned to normal during transfer to the nearest cardiology department (Fig. 1b).

Transthoracic echocardiography showed severe hypokinesis of the posteroinferior wall from base to apex with altered left ventricular systolic function (ejection fraction = 30%) and moderate ischemic mitral insufficiency.

Laboratory tests showed the following: serum troponin I, 4.7 ng/l; creatine kinase–myocardial band (MB), 2096 mmol/l; brain natriuretic peptides (BNP) 110 ng/ml; and no abnormalities in electrolytes, renal function, and routine blood tests.

Coronarography was performed 24 hours later and showed mild and proximal stenosis of his left anterior descending artery (LAD) without evidence of ruptured vulnerable plaque, and severe proximal circumflex artery occlusion that required balloon dilatation and artery stenting. The result was middling with low coronary blood flow after percutaneous transluminal angioplasty: Thrombolysis in Myocardial Infarction Grade (TIMI) Flow II.

On the second day of hospitalization, he presented a cardiogenic shock concomitant with severe ventricular arrhythmia. The rhythm disorder was reduced by external electric shock. The reappearance of the ST segment elevation in lateral territory with persistent hemodynamic instability imposed treatment by a dobutamine infusion and urgent coronarography which revealed circumflex artery in-stent restenosis. An attempt to open the artery by balloon angioplasty was unsuccessful. A systolic electro drive probe was placed. On day 4, he presented an altered neurological status requiring intubation, mechanical ventilation, and sedation. Cerebral imaging showed massive inoperable intraparenchymal hemorrhage. The outcome was fatal on the sixth day of hospitalization (Additional file 1).

### Case 2

A previously healthy 45-year-old North African man was brought to our emergency room complaining of generalized rash a few minutes after manipulating some plants in his garden. He immediately received 4 mg of dexamethasone intravenously for suspicion of anaphylactic reaction. Half an hour later, he developed severe retrosternal pain radiating to his left arm accompanied with epigastric discomfort. His vital signs were stable: his systolic blood pressure was 110 mmHg, his heart rate was 98 beats per minute, and his oxygen saturation was 99%.

**Fig. 2:**
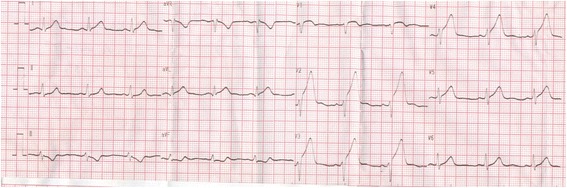
Electrocardiogram showing ST elevation in anterior leads with ST depression in inferior wall

An ECG showed ST elevation in anterior leads with ST depression in inferior wall (Fig. 2). Upon arrival of the Emergency Medical Service (EMS) team, he was conscious and hemodynamically stable with regression of the hives. KS was immediately suspected. Anti-ischemic treatment was administered: heparin (50 mg intravenously administered), aspirin (250 mg intravenously administered) and clopidogrel (600 mg administered orally). He was transferred to the nearest catheterization room for primary angioplasty.

**Fig. 3:**
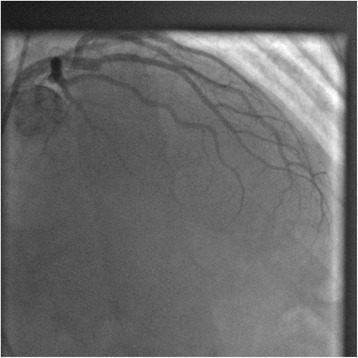
Coronarography showing total *mid-left* anterior descending artery occlusion (Thrombolysis in Myocardial Infarction Grade Flow 0)

**Fig. 4:**
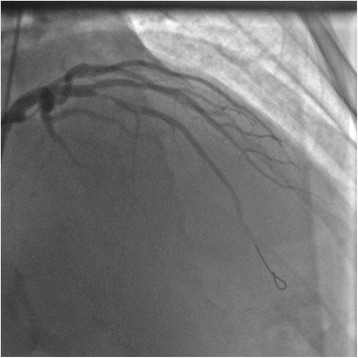
Partial opening of *left* anterior descending artery after balloon inflation at the occlusion site (Thrombolysis in Myocardial Infarction Grade Flow I)

**Fig. 5:**
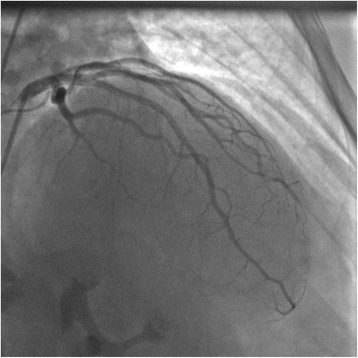
Reopening of *left* anterior descending artery after thrombo-aspiration and stenting (Thrombolysis in Myocardial Infarction Grade Flow III)

A coronary angiogram revealed total occlusion of his mid-LAD. His left circumflex artery and his right coronary artery were normal (Fig. 3). Balloon inflation at the occlusion site allowed restoration of blood flow (TIMI Flow I; Fig. 4). The lesion was very thrombotic, so the interventional cardiologist performed thrombo-aspiration and intracoronary abciximab injection, which was completed by a bare metal stent (3×18 mm) implantation. The final angiography result was good with TIMI Flow III (Fig. 5).

The outcome was favorable and he was discharged from hospital 5 days later on adequate treatment with close follow-up (Additional file 1).

## Discussion

In the light of the above cases, we suggest that anaphylactic reaction was the triggering factor of ACS which was confirmed by the association of an angina pain, ECG modifications, increased levels of cardiac troponin, and the occurrence of allergic symptoms at the same time.

The association of allergic reaction and ACS was first reported in 1951 by Pfister but it was not described again until 1991 when Kounis published the first description of this syndrome. This entity has been identified as “the coincidental occurrence of chest pain and allergic reactions accompanied with clinical and laboratory findings of classic angina pectoris caused by inflammatory mediators released during the allergic insult” [1]. The heart condition occurs in a considerable number of patients during episodes of anaphylaxis [2]; there are three variants of KS. The first is observed in patients with no previous heart condition in which the inflammatory cascade triggered by the allergic insult causes a coronary vasospasm accompanied with elevated levels of myocardial damage markers. The second is observed in patients with pre-existing atheromatous disease in whom the release of these mediators would also produce a coronary vasospasm, which occurs with rupture of the atheromatous plaque, and the third is stent thrombosis [2–4].

The patients described here presented coronary disease and one had cardiovascular risk factors which allow us to classify them in the second variant of KS types.

The term “cardiac anaphylaxis” refers to the functional and metabolic cardiac changes caused by the release of histamine and metabolites arising from the arachidonic acid cascade following a serious allergic insult. Furthermore, mast cells are the major actors of this inflammatory process by liberating inflammatory mediators locally and into the systemic circulation [2].

These mediators have several cardiovascular activities that induce coronary vasoconstriction and platelet activation leading to plaque erosion and rupture. They also induce tachycardia, dysfunctional ventricular contractility, and blockade of atrioventricular conduction. Another pathogenic mechanism proposed for ACS complicating anaphylaxis is prolonged hypotension, especially in patients with critical coronary stenoses, in whom hypotension may precipitate an abrupt reduction of coronary flow and provoke myocardial ischemia in the stenotic artery’s territory [2]. Management of KS consists of restoring myocardium revascularization in conjunction with the treatment of the allergic reaction. The difficulty lies in the fact that the treatment of either of the two associated entities may worsen the other injury. Until now, guidelines for the treatment of KS are lacking and most of the recommendations published on the efficacy and safety of the treatment is based on individual case reports.

For the treatment of the allergic reaction, both H1 and H2 antihistamines, such as diphenhydramine (1 to 2 mg/kg) and ranitidine (1 mg/kg) can be used. Bolus administration can precipitate hypotension and compromise coronary flow; therefore, these drugs should be given slowly.

Corticoids have an important role in the treatment of anaphylaxis; despite reported side effects in ACS (corticosteroids may impair wound healing causing myocardial wall thinning and cardiac aneurysms), their use in KS is probably safe and appropriate.

Fluid resuscitation is the mainstay treatment in the management of distributive shock. Its use in such circumstances should take into consideration the patient’s specific clinical conditions such as ejection fraction, acute pulmonary edema, and hemodynamic instability.

Epinephrine is the medication of choice to relieve the life-threatening symptoms of anaphylaxis; but, it may aggravate myocardial ischemia and induce coronary vasospasm and arrhythmias in KS especially if administrated intravenously. Thus, an intramuscular route is preferred in a dose of 0.2 to 0.5 mg each 5 to 15 minutes, until the resolution of symptoms or the appearance of epinephrine side effects [3, 5].

Management of ACS should follow the recent guidelines of the American College of Cardiology Foundation and the American Heart Association in the absence of specific guidelines for KS. Primary percutaneous coronary intervention is the preferred reperfusion strategy if it can be performed by an experienced team and within guideline-mandated times (i.e. 120 min of first medical contact). Otherwise, fibrinolytic therapy should be considered [6].

Pharmacological treatment of myocardial revascularization includes adjunctive antithrombotic therapy: acetylsalicylic acid (ASA) and a P2Y12 receptor inhibitor [6].

Aspirin is able to induce an important antithrombotic effect in patients with ACS but there is a risk that it can induce or worsen an anaphylactic reaction. An additive antiplatelet effect is observed with a loading dose of a P2Y12 receptor inhibitor which should be given as early as possible. These molecules do not have a harmful effect on the associated anaphylactic reaction. For these reasons, aspirin should be given under close monitoring, in the absence of previous history of allergic reaction to ASA, and after the treatment of anaphylactic reaction. In case of allergy, this drug must be avoided and replaced by alternative antiplatelet agents [3, 5].

Medical treatment also includes intravenously administered nitroglycerin which is indicated in the first 48 hours after an ACS for treatment of persistent ischemia, heart failure, or hypertension [5]. Although nitrates may induce an allergic reaction, this molecule seems safe in KS when the hemodynamic status is satisfactory [6].

The use of beta-blockers in ACS is very beneficial outside contraindications (heart failure, cardiogenic shock, conduction abnormalities, and so on). In KS, these agents may interfere with the use of epinephrine which is the basis of treatment of anaphylaxis. Furthermore, they can cause a subsequent coronary spasm due to an unopposed activity of α-adrenergic receptors. Consequently, beta-blockers are contraindicated in KS. Opioids, as powerful analgesic and anxiolytic agents, are indicated to relieve the chest pain but their use may induce massive mast cell degranulation which may aggravate the anaphylactic reaction. They should hence be given carefully in patients with KS [5, 6].

Similar cases to the ones reported in this article were rare in the literature. It is important to evoke the diagnosis of KS when necessary and to understand its physiopathological mechanisms in order to modify or adapt the management of ACS to get the optimal result and avoid aggravation of the patient’s condition.

## Conclusions

KS is the association of ACS with an anaphylactic reaction. Emergency physicians must be aware of this entity and suspect it whenever they face patients with both anaphylactic reaction and angina symptoms. An immediate diagnosis is necessary to urgently initiate adequate treatment.

The treatment of KS is challenging because it requires urgent management of both anaphylaxis and cardiac infarction. The difficulty lies in the fact that the treatment of either of the two associated entities may worsen the other injury.

Many questions about the optimal treatment of KS remain unanswered. The use of an international register could enable the collection of the necessary information to establish a standardized therapeutic protocol for this entity.

## Additional file

Additional file 1: Timeline. (DOCX 19 kb)

## Abbreviations

ACS: Acute coronary syndrome
ASA: Acetylsalicylic acid
ECG: Electrocardiogram
EMS: Emergency Medical Service
GCS: Glasgow Coma Scale
KS: Kounis syndrome
LAD: Left anterior descending artery
TIMI: Thrombolysis in Myocardial Infarction Grade
