# Supplementary material for: Management of Kounis syndrome: two case reports
Source: J Med Case Rep. 2017 May 23;11:145. doi: 10.1186/s13256-017-1310-7 (PMC5440976; doi:10.1186/s13256-017-1310-7)
Supplement: Additional file 1: — Timeline. (DOCX 19 kb) [file 13256_2017_1310_MOESM1_ESM.docx]

**CASE 1:**

60-year-old north-African man

Allergy to penicillin, type 2 diabetes, hypertension, ischemic stroke 4 years ago without sequels Medication included Captopril, Furosemid and Metformin.

Cardiogenic shock concomitant with ventricular tachycardia and ST elevation in lateral territory: cardioversion, inotropic drugs, Coronarography showed restenosis of circumflex with failure of angioplasty.

Neurological alteration: the patient was intubated and cerebral MRI showed an intra-parenchymal hemorrhage of great abundance.

Death (day 6)

Coronarography: severe circumflex artery stenosis which required balloon dilatation and artery stenting.

Cedation of pain and regression of ST elevation in ECG

Diagnosis

Examination

IV Hydrocortisone Hemisuccinate 200 mg, PO diphenhydramine 50mg, IV Heparin: 50mg, IV Aspirin 250mg, Oral Clopidogrel 300 mg. Fibrinolysis IV Streptokinase 1.5M during 45 min

Acute coronary syndrome with ST elevation concomitant with an anaphylaxis reaction

ECG: ST segment elevation of 3 mm in inferior leads and reciprocal change in the antero-lateral wall

GCS of 14/15, generalized urticaria, Vital signs stable

At Presentation

Chest pain, dizziness, pruritus, warmth, flushing, dyspnea and an altered mental status

T-30min

**T0**

**(11/05/2015 at 12pm)**

T+30min

T + 40min

T +45min

T +1h30

T +24h

T +48h

Day 4

Auto medication by 1 g amoxicillin for a dry cough

**CASE 2:**

45-year-old north-African male

No medical history or coronary disease risk factors

T0

T+10min

T+30min

T+35min

T +40min

T +120min

Until day 5

Generalized rash few minutes after manipulating some plants

At Presentation

Stable vital signs

IV Heparin: 50mg, IV Aspirin 250mg, Oral Clopidogrel 600 mg

Examination

Diagnosis

Coronarography: an occlusion of the anterior intervenricular artery. Successful angioplasty

Transfer to the nearest catheterization room

Hospitalization without further complication

Good evolution

Discharge (Day 5)

IV Hydrocortisone Hemisuccinate 200 mg

**Anaphylaxis reaction**

Severe retrosternal chest pain radiating to the left arm

Stable vital signs

ECG: showed ST elevation in anterior leads with ST depression in inferior wall

Examination

Diagnosis

**Acute coronary syndrome with ST elevation concomitant with an anaphylaxis reaction**
